# Time trends and projected obesity epidemic in Brazilian adults between 2006 and 2030

**DOI:** 10.1038/s41598-022-16934-5

**Published:** 2022-07-26

**Authors:** José Matheus Estivaleti, Juan Guzman-Habinger, Javiera Lobos, Catarina Machado Azeredo, Rafael Claro, Gerson Ferrari, Fernando Adami, Leandro F. M. Rezende

**Affiliations:** 1 Department of Preventive Medicine, Escola Paulista de Medicina, Universidade Federal de São Paulo, Sao Paulo, Brazil; 2 Facultad de Ciencias, Especialidad Medicina del Deporte Y La Actividad Física, Universidad Mayor, Santiago, Chile; 3 Datrics, 7500000 Santiago, Chile; 4 Faculdade de Medicina, Universidade Federal de Uberlândia, Uberlândia, Brazil; 5 Departamento de Nutrição, Escola de Enfermagem, Universidade Federal de Minas Gerais (UFMG), Minas Gerais Belo Horizonte, Brasil; 6 Universidad de Santiago de Chile (USACH), Escuela de Ciencias de la Actividad Física, El Deporte y la Salud, Santiago, Chile; 7 Laboratório de Epidemiologia e Análise de Dados, Centro Universitário FMABC, Santo André, São Paulo, Brasil

**Keywords:** Diseases, Risk factors

## Abstract

We examined time trends and projected obesity epidemic in Brazilian adults between 2006 and 2030 by sex, race/skin color, educational attainment, and state capitals. Self-reported body weight and height of 730,309 adults (≥ 18 years) from the Vigitel study were collected by telephone interview between 2006 and 2019. A multinomial logistic regression model was used to predict the prevalence of body mass index (BMI) categories as a function of time by 2030. The prevalence of obesity increased from 11.8% in 2006 to 20.3% in 2019. The projected prevalences by 2030 are estimated to be 68.1% for overweight, 29.6% for obesity, and 9.3% for obesity classes II and III. Women, black and other minority ethnicities, middle-aged adults, adults with ≤ 7 years of education, and in Northern and Midwestern capitals are estimated to have higher obesity prevalence by 2030. Our findings indicate a sustained increase in the obesity epidemic in all sociodemographic subgroups and across the country. Obesity may reach three out of 10 adults by 2030.

## Introduction

Studies have documented the increasing obesity epidemic across the globe^1^. Between 1975 and 2016, the age-adjusted prevalence of obesity (i.e., defined as body mass index—BMI ≥ 30 kg/m^2^) tripled worldwide, reaching 16% or 650 million adults in 2016^1^. The World Health Organization (WHO) Plan of Action for the Control of Noncommunicable Diseases aims to reduce by half the increase (slope) in the prevalence of obesity by 2025^2^. However, projections suggest this goal may not be achieved^3^. In Brazil, epidemiological studies have also shown an increase in the prevalence of obesity in adults from 12.5% in 2002 to 25.9% in 2019^4,5^. Overall, the prevalence of obesity has been higher in adults of lower socioeconomic levels and educational attainment^4,5^.

Recently, a few studies conducted in high-income countries, such as the United States^6^ and England^7^, have projected their national prevalence of obesity to inform local actions aimed to control the obesity epidemic^6,7^. In low- and middle-income countries, such as Brazil, projections of future obesity epidemic are scarce, despite its potential to inform public health policies and decision-makers. In Brazil, the Surveillance System for Risk and Protective Factors for Chronic Diseases by Telephone Survey (Vigitel) has conducted yearly since 2006^8^. Thereafter, over 50 thousand adults from 27 state capitals and the Federal District (hereinafter defined as geographic units) have annually reported their body weight, height, and demographic and socioeconomic characteristics, enabling the study of the current and future distribution of obesity in the Brazilian population over time^8^.

In this study, we examined time trends (2006–2019) and projected (2020–2030) the prevalence of BMI categories in Brazilian adults between 2006 and 2030, according to sex, age group, race/skin color, educational attainment, and geographic units.

## Methods

### Study design, data source, and sample

Vigitel is an annual telephone survey conducted between 2006 and 2019 to monitor health indicators in adults aged ≥ 18 years from 26 state capitals and the Federal District. A sample of approximately 2000 individuals was interviewed in each city per year, so that all indicators included in the system could be assessed with a 95% confidence interval (CI) and a sample error of two percentage points. The sampling process was conducted in two stages. The first one consisted of sampling 5000 landlines per city (from lists of household telephone numbers provided by the main operators in the country), randomly reorganized into 25 replicates (200 numbers). Each landline selected was contacted up to six times on different days and times (from nine am to nine pm, including weekends and holidays) to assess participants’ eligibility. Out-of-service, non-residential, and non-answering lines were considered ineligible. In the second stage, one adult from each household was randomly selected and invited to participate in the study. The weighting factors provided by the Brazilian Ministry of Health equate the distribution of the population interviewed by Vigitel to that predicted for the entire adult population in each municipality, estimated in two stages. The first stage aimed to correct the unequal probability of selecting households with more than one landline or resident, and the second stage to balance the distribution of the interviewed population in each city (by sex, age and educational attainment) to the entire population (based on official projections for each year via the Rake procedure)^9^. Vigitel was approved by the National Research Ethics Commission, and the identified databases are available at: http://svs.aids.gov.br/download/Vigitel/. All aspects of the study were in accordance with the Declaration of Helsinki. From 2006 and 2019, 730,309 adults (aged ≥ 18 years) responded to the Vigitel questionnaire, but 64,088 had missing data for self-reported weight and height. We used Hot Deck imputation method for non-survey response based on municipality, sex, age and education attainment^10^, producing a final analytical sample of 730,309 adults from Vigitel 2006–2019.

### Statistical analysis

#### Time trends and projected prevalences of BMI categories

We examined time trends in the prevalence of BMI categories between 2006 and 2019 according to sociodemographic characteristics (sex, age group, race/skin color, education attainment) and geographic units. BMI categories were defined per the WHO classification: underweight or normal weight (BMI < 25 kg/m^2^), pre-obesity (25 to < 30 kg/m^2^), obesity class I (30 to < 35 kg/m^2^), and obesity classes II and III (≥ 35 kg/m^2^)^11^. We also performed time trend analysis considering the definitions of overweight (≥ 25 kg/m^2^) and obesity (≥ 30 kg/m^2^). We performed simple Poisson and linear regression models to estimate the relative (prevalence ratio—PR) and absolute (prevalence differences—in percentage points—p.p.) increases in the prevalence of overweight, obesity and obesity classes II and III between 2006 and 2019.

A simple multinomial logistic regression model was used to predict the prevalence of each BMI category over time from 2006 through 2030. This model ensured the prevalence of all BMI categories adds up to 100% each year, and enables the estimation of a nonlinear trend in the prevalence of BMI categories^6^. In addition, it implicitly considers the demographic composition of the population and the factors contributing to BMI changes over time (e.g., consumption of ultra-processed foods and physical inactivity)^6^. Thus, unlike studies estimating the causal effects of exposures on obesity risk, studies predicting BMI over time require no confounding control^6^. Therefore, our simple multinomial logistic regression model included only BMI category (dependent variable) and time (independent variable). Same methodological approach has been used a previous study on projected obesity in US^6^.

Regression models were conducted according to sex (men or women), age groups (18–34, 35–54 or ≥ 55 years), race/skin color (white or blacks and other minority ethnicities), educational attainment (0–7, 8–11 or ≥ 12 years of education) and geographic units (26 state capitals and the Federal District) independently, considering the Vigitel sample weights. We examined time trends (2006–2019) and projected (2020–2030) prevalence of each BMI category, as well as the prevalence of overweight (BMI ≥ 25 kg/m^2^), obesity (BMI ≥ 30 kg/m^2^) and obesity classes II and III (BMI ≥ 35 kg/m^2^).

#### Assessment of prediction accuracy

The accuracy of the prediction model was evaluated via a simple multinomial logistic regression model for BMI categories (dependent variable) and time (independent variable) from 2006 to 2013. The predicted prevalence of each BMI category from 2014 to 2019 was estimated according to sex, and compared with the corresponding observed prevalence for that same year-sex. Model accuracy was measured via two metrics. First, we calculated the coverage probability, in which the 95% CI of each predicted prevalence between 2014 and 2019 were evaluated to ascertain whether they included the observed prevalence for that same year-sex stratum. Second, we evaluated whether the difference between predicted and the observed prevalences was lower than 10% (10% relative error).

### Ethics approval

Vigitel was approved by the National Research Ethics Commission, and the identified databases are available at: http://svs.aids.gov.br/download/Vigitel/.

## Results

### Time trends prevalence of overweight, obesity and obesity class II and III between 2006 and 2019

**Figure 1:**
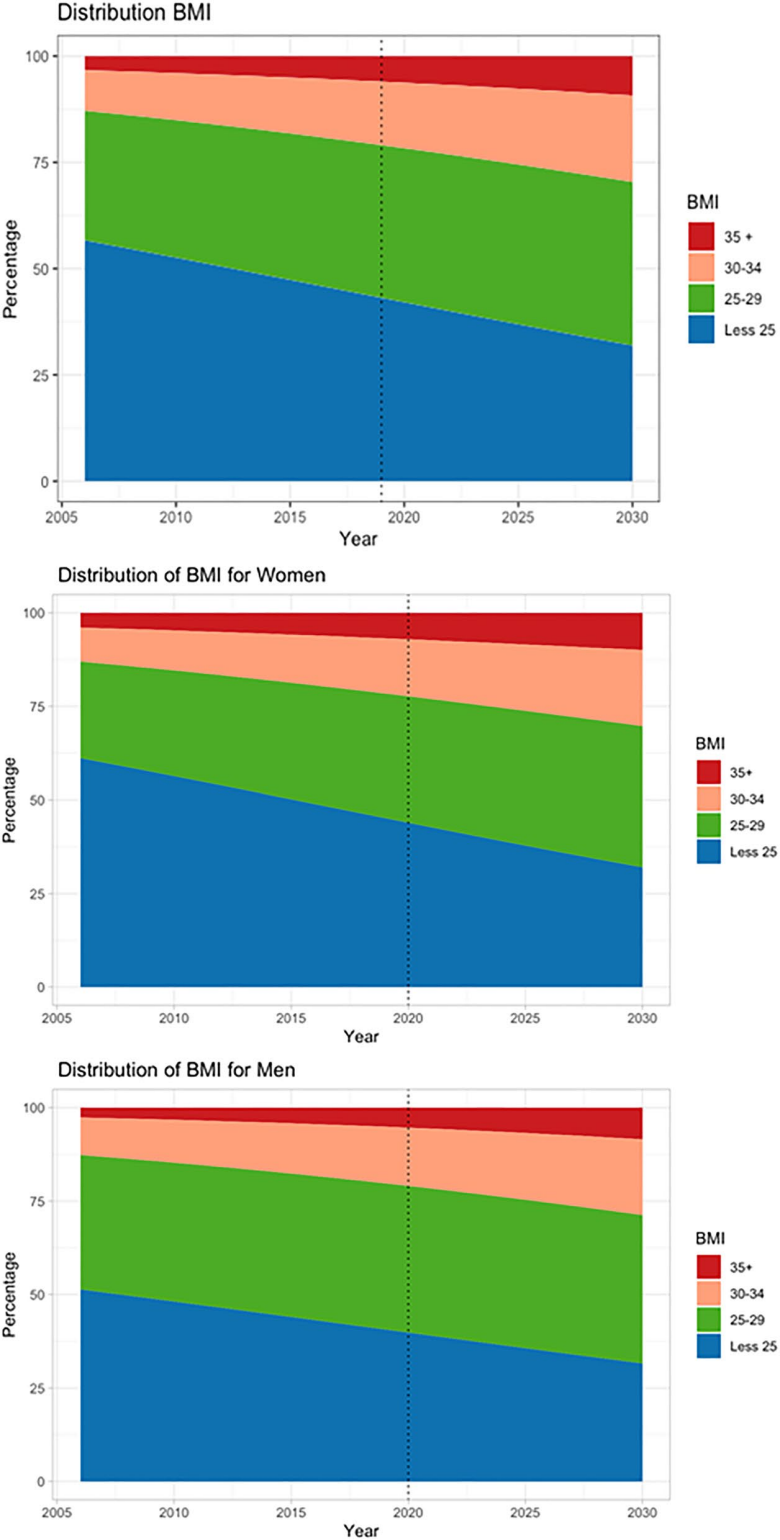
Time trends and projected prevalence of body mass index categories in Brazilian adults between 2006 and 2030 according to sex.

In 2006, the prevalence of overweight, obesity and obesity classes II and III in Brazilian adults were 30.9%, 8.6%, and 3.2%, respectively. In 2019, the prevalences reached 35.1% for overweight, 14.6% for obesity and 5.7% for obesity classes II and III (Fig. 1 and Table 1).

**Table 1:** Time trends prevalence of overweight, obesity, and obesity classes II and III in Brazilian adults between 2006 and 2019, according to sociodemographic characteristics.

**BMI categories/sociodemographic subgroups**	**Prevalence (95% CI)**				**Prevalence ratio (95% CI)**		**Prevalence difference (95% CI)**	
	**2006**		**2019**					
**Overweight (≥ 25 kg/m**^**2**^**)**								
Overall	42.6	(41.8 to 43.5)	55.4	(54.4 to 56.3)	1.30	(1.26 to 1.33)	12.7	(11.5 to 14.0)
Men	47.5	(46.1 to 48.9)	57.1	(55.6 to 58.7)	1.20	(1.16 to 1.25)	9.6	(7.5 to 11.7)
Women	38.5	(37.4 to 39.6)	53.9	(52.7 to 55.0)	1.40	(1.35 to 1.45)	15.4	(13.8 to 17.0)
Younger adults (18–34 years)	30.3	(29.0 to 31.6)	44.9	(43.1 to 46.8)	1.48	(1.40 to 1.57	14.6	(12.4 to 16.9)
Middle-aged (35–54 years)	51.4	(50.0 to 52.8)	62.3	(60.1 to 63.7)	1.21	(1.17 to 1.26)	10.9	(8.9 to 12.9)
Older adults (55 + years)	54.4	(52.6 to 56.2)	61.5	(60.3 to 62.8)	1.13	(1.09 to 1.18)	7.2	(4.9 to 9.4)
Blacks and other ethnical minorities	42.8	(41.7 to 43.9)	56.5	(55.2 to 57.7)	1.32	(1.28 to 1.37)	13.7	(12.0 to 15.4)
Whites	42.4	(41.1 to 43.8)	53.8	(52.3 to 55.3)	1.27	(1.22 to 1.32)	11.4	(9.3 to 12.4)
0–7 years of education	49.7	(48.0 to 51.3)	61.1	(59.0 to 63.2)	1.23	(1.17 to 1.29)	11.4	(8.7 to 14.1)
8–11 years of education	39.4	(38.2 to 40.6)	55.0	(53.6 to 56.4)	1.40	(1.34 to 1.45)	15.6	(13.8 to 17.4)
12 + years of education	37.3	(35.7 to 39.0)	52.2	(50.6 to 53.9)	1.40	(1.32 to 1.48)	14.9	(12.6 to 17.3)
**Obesity (≥ 30 kg/m**^**2**^**)**								
Overall	11.8	(11.2 to 12.4)	20.3	(19.5 to 21.0)	1.72	(1.62 to 1.83)	8.5	(7.6 to 9.4)
Men	11.4	(10.5 to 12.3)	19.5	(18.3 to 20.7)	1.71	(1.55 to 1.89)	8.1	(6.6 to 9.6)
Women	12.1	(11.4 to 12.9)	21.0	(20.0 to 21.9)	1.73	(1.60 to 1.86)	8.8	(7.6 to 10.0)
Younger adults (18–34 years)	7.5	(6.7 to 8.3)	15.5	(14.2 to 16.9)	2.08	(1.82 to 2.38)	8.1	(6.5 to 9.6)
Middle-aged (35–54 years)	14.2	(13.3 to 15.2)	23.6	(22.4 to 24.9)	1.66	(1.53 to 1.81)	9.4	(7.8 to 11.0)
Older adults (55 + years)	17.1	(15.7 to 18.5)	22.7	(21.7 to 23.8)	1.33	(1.21 to 1.47)	5.7	(3.9 to 7.5)
Blacks and other ethnical minorities	12.1	(11.4 to 12.8)	21.5	(20.5 to 22.5)	1.78	(1.65 to 1.92)	9.4	(8.2 to 10.7)
Whites	11.4	(10.5 to 12.3)	18.5	(17.4 to 19.6)	1.62	(1.47 to 1.80)	7.1	(5.7 to 8.5)
0–7 years of education	16.0	(14.9 to 17.3)	25.1	(23.4 to 26.9)	1.56	(1.41 to 1.73)	9.1	(6.9 to 11.2)
8–11 years of education	9.8	(9.2 to 9.6)	20.3	(19.2 to 21.4)	2.07	(1.90 to 2.25)	10.5	(9.2 to 11.7)
12 + years of education	8.6	(7.8 to 9.6)	17.2	(16.0 to 18.5)	1.99	(1.76 to 2.26)	8.6	(7.8 to 9.5)
**Obesity class II and III (≥ 35 kg/m**^**2**^**)**								
Overall	3.2	(2.9 to 3.5)	5.7	(5.3 to 6.1)	1.76	(1.56 to 1.99)	2.4	(1.9 to 3.0)
Men	2.6	(2.2 to 3.1)	4.7	(4.2 to 5.4)	1.80	(1.44 to 2.25)	2.1	(1.3 to 2.9)
Women	3.7	(3.3 to 4.2)	6.5	(5.9 to 7.0)	1.74	(1.51 to 2.01)	2.7	(2.0 to 3.4)
Younger adults (18–34 years)	2.1	(1.7 to 2.6)	3.8	(3.2 to 4.5)	1.81	(1.38 to 2.38)	1.7	(0.9 to 2.5)
Middle-aged (35–54 years)	3.7	(3.2 to 4.3)	6.9	(6.2 to 7.8)	1.87	(1.56 to 2.24)	3.2	(2.3 to 4.2)
Older adults (55 + years)	4.8	(4.0 to 5.7)	6.6	(6.0 to 7.3)	1.39	(1.14 to 1.70)	1.9	(0.8 to 2.9)
Blacks and other ethnical minorities	3.2	(2.8 to 3.5)	6.2	(5.7 to 6.8)	1.97	(1.70 to 2.29)	3.1	(2.4 to 3.8)
Whites	3.3	(2.8 to 3.9)	4.8	(4.3 to 5.4)	1.48	(1.20 to 1.82)	1.6	(0.8 to 2.4)
0–7 years of education	5.0	(4.3 to 5.8)	7.6	(6.7 to 8.7)	1.52	(1.25 to 1.86)	2.6	(1.4 to 3.9)
8–11 years of education	2.2	(1.9 to 2.6)	5.7	(5.1 to 6.3)	2.55	(2.13 to 3.06)	3.4	(2.8 to 4.1)
12 + years of education	2.2	(1.8 to 2.7)	4.4	(3.8 to 5.1)	2.01	(1.55 to 2.61)	2.2	(1.4 to 3.0)

Abbreviation: CI, confidence interval; Kg/m^2^, kilograms per meters squared.

The prevalences of overweight, obesity and obesity classes II and III increased in all sociodemographic subgroups between 2006 and 2019 (Table 1). However, speed and extent of weight gain varied by sex, age group, race/skin color and educational attainment. We observed a higher relative increase in the prevalence of overweight between 2006 and 2019 in women (PR 1.40, 95% CI 1.35–1.45), young adults (PR 1.48, 95% CI 1.40–1.57), blacks and other minority ethnicities (PR 1.32, 95% CI 1.28–1.37), and adults with 8–11 years of education (PR 2.07, 95% CI 1.90–2.25). Similar pattern was observed for the increase in the prevalence of obesity by subgroups. For obesity classes II and III, the relative increase in the same period was higher in men (PR 1.80, 95% CI 1.44–2.25), middle-aged adults (PR 1.87, 95% CI 1.56–2.24), blacks and other minority ethnicities (PR 1.97, 95% CI 1.70–2.29), and adults with 8–11 years of education (PR 2.55, 95% CI 2.13–3.06). Absolute differences in the prevalence of overweight, obesity and obesity classes II and II between 2006 and 2019 are displayed in the Table 1.

### Projected prevalence of overweight, obesity and obesity class II and III by 2030

**Figure 2:**
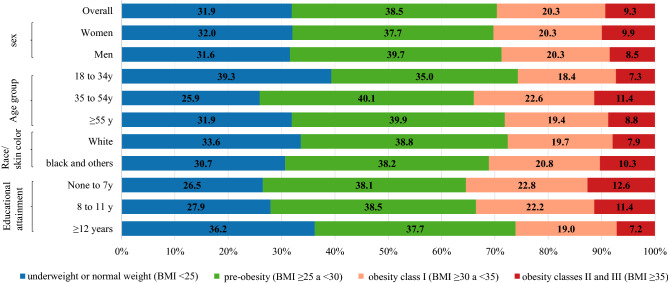
Projected prevalence of underweight or normal weight, pre-obesity, obesity and obesity classes II and III in Brazilian adults by 2030 according to sociodemographic characteristics.

The projected prevalences by 2030 are estimated to be 68.1% for overweight, 29.6% for obesity and 9.3% for obesity classes II and III (Fig. 2). The prevalence of obesity is estimated to be 30.2% in women and 28.8% in men. Middle-aged adults, blacks and other minority ethnicities, and adults with lower educational attainment (none to seven years) are also estimated to have higher prevalences of obesity by 2030 (Fig. 2).

Northern and Midwestern capitals are estimated to have the highest prevalence of obesity by 2030; namely, Manaus (35.8%; 95% CI 31–40.6); Cuiabá (34.9%; 95% CI 30.7–39.1), and Rio Branco (32.8%; 95% CI 29.5–39.8). The capitals with the lowest prevalence of obesity are estimated to be Florianópolis (23.0%; CI 95% 19.6–26.5), Palmas (23.8% 95% CI 19.8–27.8), and Curitiba (24.9% 95% CI 21.5–28.4).

In 2030, a quarter of the adult population may be living with obesity (prevalence of obesity ≥ 25%) by 2030 in 24 (88.8%) out of the 27 geographic units. The prevalence of obesity classes II and III is estimated to be higher than 10% in 8 (29.6%) out of 27 geographic units (Fig. S1 and Table S1).

### Predictive accuracy of the model

Our model had a 21% coverage probability (i.e., the proportion of 95% CI for the predicted obesity prevalence between 2014 and 2019 which included the observed prevalence for the same sex- and year-stratum). All projected prevalence from 2014 to 2019 showed a relative error lower than 10% (10% relative error) in relation to the observed prevalence. Our model showed a 54% accuracy when considered a 5% relative error (Table S2).

## Discussion

In this study, we used data from 730,309 participants from Vigitel to examine time trends and projected obesity epidemic in Brazil between 2006 and 2030. Between 2006 and 2019, we observed a 30% increase in the prevalence of overweight (from 42.6 in 2006 to 55.4% in 2019), a 72% increase in the prevalence of obesity (from 11.8 to 20.3%) and a 76% increase in the prevalence of obesity classes II and III (from 3.2 to 5.7%). The prevalences of BMI categories by 2030 are estimated to be 68.1% for overweight, 29.6% for obesity and 9.3% for obesity classes II and III.

The obesity epidemic is a global public health concern. In 2016, approximately 1.9 billion adults were living with obesity, and an increasing time trend has been observed in almost every country in the world^1^. Studies on the projected future trajectories of obesity prevalence are scarce in low- and middle-income countries, despite its potential to inform the need for preventive strategies and preparedness of health systems to cope with obesity consequences. The World Obesity Atlas 2022 have recently estimated that 1 in 5 women and 1 in 7 men will live with obesity by 2030^12^. The global projected prevalence by 2030 is estimated to be 17.5% (approximately, 1 billion people) for obesity and 5.7% (333 million people) for obesity classes II and III. In Brazil, their projected prevalence of obesity is estimated to be 33% for women and 25% for men by 2030, which are similar to our findings (women 30.2%; men 28.8%). Although these findings indicate an increasing obesity epidemic in Brazil, the Brazilian figures are still lower than other countries in Americas and worldwide. By 2030, 1 in 3 men (34.4%) and almost two-fifths of women (39.7%) living in Americas region are predicted to have obesity. The 10 highest projected prevalence of obesity in the Americas region ranged from 47% in US to 32% in Dominican Republic. In the global rank, Brazil is also not listed in the top 20 countries with highest projected prevalence of obesity by 2030 (women: 69% in American Samoa to 50% in Turkey; Men: 67% in Nauru to 39% in Canada). Nonetheless, the increasing prevalence of obesity in Brazil is a concern as it will increase the burden of non-communicable diseases (NCDs) and its associated costs to the Brazilian Unified Health System^13^.

Our findings indicate socioeconomic disparities in time trends and projected prevalence of obesity epidemic in Brazil. Data from 103 countries has also shown that as countries develop economically, overweight rates rise, affecting poorer individuals more markedly^14^. Of note, we observed a higher relative increase in the prevalence of overweight between 2006 and 2019 in women, young adults, blacks and other minority ethnicities, and adults with 8–11 years of education. Similar results were observed for absolute increases in the prevalence of obesity. By 2030, 6 out of 10 adults with lower educational attainment (< 7 years of education) are estimated to be living with overweight. Adults with lower educational attainment and blacks and other minority ethnicities may have an even higher prevalence of obesity classes II to III by 2030, compared to its respective counterparts. In Brazil, educational attainment is a good proxy of socioeconomic status. People without access to formal education or those with < 7 years of education (elementary school only) are more likely to have lower socioeconomic status and worse health outcomes^15,16^. These results corroborate the projected prevalence of obesity in the United States by 2030: 55.6% of participants with household income below $20.000/year will be living with obesity *vs* 41.7% in participants with higher household income (≥ $50.000/year). Lower educational attainment was also associated with a higher prevalence of obesity classes II to III in US^17^. These findings suggest that public policies aimed at mitigating obesity inequalities are imperative.

Obesity is a major risk factor for several NCDs, such as cardiovascular diseases, diabetes, and several types of cancer^18,19^. In 2019, NCDs were responsible for 55% of the 738.371 deaths in Brazil^20^, of which, 56.1% or 173.207 occurred in adults aged 30–69 years and, therefore, are premature and preventable (in principle). The increasing obesity epidemic has contributed to the increasing burden of cancer in Brazil. Approximately 15,000 cancer cases per year are attributable to high BMI in Brazil, and projections suggest that this number could surpass 29.000 cases by 2025^18^, The worldwide increase in obesity will impact the rise of other NCDs. Projections indicate that type 2 diabetes will affect at least half a billion people by 2030^21^. For each 4 kg/m^2^ increase in BMI there is a 26% to 56% increase in the risk of ischemic heart disease^22^. In Brazil, overweight and obesity has caused more than 30,000 deaths per year from cardiovascular diseases, cancers and respiratory diseases^23^.

Our study has important public health implications. Projected obesity epidemic in Brazil — a middle-income country with limited health care resources — reinforce that primary prevention is pivotal to change obesity trajectories in the country. Our findings also highlight the importance of obesity prevention strategies focusing the whole population, surveillance systems, and prevention research to better evaluate and design public health strategies^24^. Moreover, our results showed that obesity affect more socioeconomically deprived groups, whose tend to have less access to healthcare and worse health outcomes^14^. Therefore, public health policies better directed at preventing obesity and reducing social inequalities may reduce the disease burden for future generations, change the predicted trend, and protect vulnerable individuals.

Our predictions assume that no major changes in obesity determinants will take place in the next years. However, since the beginning of the Coronavirus pandemic in 2020, the world has been facing an unprecedent health, economic, and social crises, which disproportionately affect poor individuals and low- to middle-income countries^25–27^. Unemployment and inflation have risen in Brazil, and austerity measures have jeopardized funding for social protection, compromising the food and nutrition security of vulnerable groups^28^. Thus, we expect that the impoverishment of the population, alongside the limited economic access to healthy food and physical activity might lead to a sharper increase in obesity rates in the near future^29^.

This study has some limitations. Weight and height were self-reported and therefore misclassification of BMI categories may have ocurred^30^. In addition, we used hot deck imputation method due to 8.7% missing data of weight or height. The use of landlines in the survey may have included adults with a higher socioeconomic level than the average population of Brazilian capitals^31,32^. However, BMI results from Vigitel have a high agreement with other nationally representative surveys in Brazil. Similarly, projected obesity epidemic based on adults living in capital cities may not reflect the entire Brazilian adult population, since they are more industrialized and economically developed than the non-capital municipalities. Our model had a moderate to good predictive accuracy.

## Conclusions

In conclusion, our results indicate an increasing obesity epidemic in Brazil. The prevalences of overweight, obesity and obesity classes II and III increased in all sociodemographic subgroups between 2006 and 2019. However, speed and extent of weight gain varied by sex, age group, race/skin color and educational attainment. We observed a higher relative increase in the obesity epidemic among women, young adults, blacks and other minority ethnicities, and adults with 8–11 years of education.

The projected prevalence of obesity may affect 3 out of 10 Brazilian adults by 2030; obesity classes II and III may affect 1 out of 10 adults. Our results also highlighted marked regional and sociodemographic inequalities in the obesity epidemic by 2030; approximately 24 out of the 27 geographic units may have more than a quarter of their population living with obesity; and prevalence of obesity classes II and III may be higher than 10% in eight out of the 27 geographic units.

## Supplementary Information

Supplementary Information 1.

Supplementary Information 2.

## Data Availability

All data generated as part of this manuscript are included as additional files in this published article.

## Abbreviations

BMI: Body mass index
CI: Confidence interval
NCD: Non-communicable diseases
WHO: World Health Organization
VIGITEL: Surveillance System for Risk and Protective Factors for Chronic Diseases by Telephone Survey
