# Supplementary figures and images for: Time trends and projected obesity epidemic in Brazilian adults between 2006 and 2030
Source: Sci Rep. 2022 Jul 26;12:12699. doi: 10.1038/s41598-022-16934-5 (PMC9315079; doi:10.1038/s41598-022-16934-5)

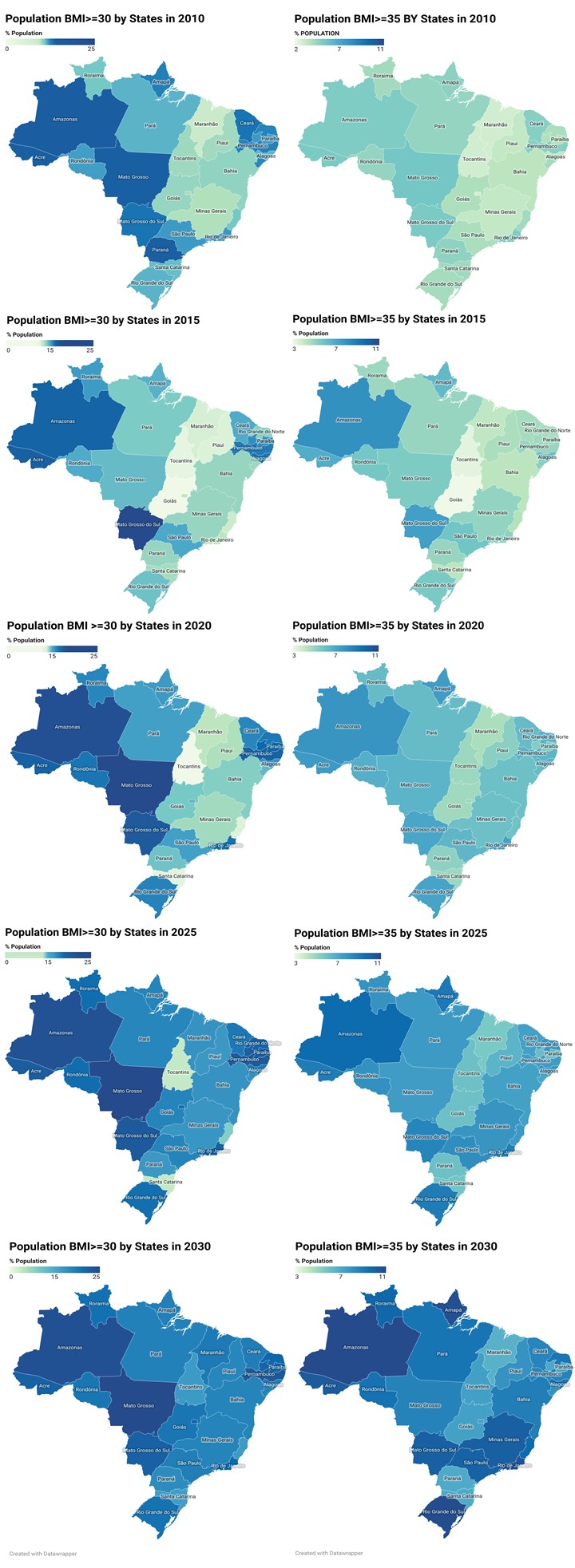

Supplement: Supplementary file 1 — Supplementary Information 1. [file 41598_2022_16934_MOESM1_ESM.jpg]
