# Supplementary material for: Time trends and projected obesity epidemic in Brazilian adults between 2006 and 2030
Source: Sci Rep. 2022 Jul 26;12:12699. doi: 10.1038/s41598-022-16934-5 (PMC9315079; doi:10.1038/s41598-022-16934-5)
Supplement: Supplementary file 2 — Supplementary Information 2. [file 41598_2022_16934_MOESM2_ESM.doc]

**SUPLEMENTARY MATERIAL**

**Table S1.** Projected capital-level prevalence of obesity and obesity class II and III by 2030 according to sex.

| **Geographic**  **location** |  | **Prevalence of Obesity (≥30 Kg/m^2^)** | | |  | **Prevalence of Obesity Class II and III (≥35 Kg/m^2^)** | | |
| --- | --- | --- | --- | --- | --- | --- | --- | --- |
|  |  | **Both sexes** | **Men** | **Women** |  | **Both sexes** | **Men** | **Women** |
| **Percentage (95% confidence interval)** | | | | | | | | |
| Aracaju |  | 26.92 (23.25-30.59) | 26.07 (20.14-32.00) | 27.58 (23.04-32.12) |  | 8.02 (5.62-10.42) | 7.59 (3.43-11.74) | 8.35 (5.51-11.18) |
| Belém |  | 28.26 (24.22-32.29) | 28.26 (21.74-34.79) | 28.03 (23.13-32.92) |  | 9.19 (6.31-12.08) | 10.9 (4.80-17.01) | 7.99 (5.18-10.79) |
| Belo Horizonte |  | 28.67 (25.07-32.28) | 30.01 (23.89-36.13) | 27.51 (23.19-31.83) |  | 10.12 (7.12-12.97) | 11.43 (5.63- 17.21) | 9.53 (6.39-12.66) |
| Boa Vista |  | 30.50 (24,94-36.07) | 34.43 (24.90-43.95) | 26.76 (20.84-32.64) |  | 9.84 (5.64-14.03 | 10.73 (3.35-18.11) | 8.95 (4.25-13.65) |
| Campo Grande |  | 32.67 (28.61-36.73) | 34.89 (28.13-41.65) | 30.59 (25.77-35.42) |  | 10.10 (7.26-12.94) | 9.86 (4.62-15.03) | 10.42 (7.13-13.70) |
| Cuiabá |  | 34.86 (30.66-39.05) | 36.09 (29.32-42.81) | 33.67 (28.58-38.76) |  | 8.60 (6.16-11.04) | 6.99 (3.60-10.37) | 10.11 (6.16-11.04) |
| Curitiba |  | 24.93 (21.49-28.36) | 30.59 (25.77-35.42) | 23.13 (18.98-27.28) |  | 6.61 (6.66-13.56) | 8.30 (4.32-12.27) | 6.12 (3.83-8.32) |
| Florianópolis |  | 23.04 (19.63-26.45) | 25.82 (20.15-31.49) | 20.52 (16.50-24.54) |  | 6.86 (4.66-9.06) | 7.51 (3.55-11.47) | 6.42 (3.90-8.94) |
| Fortaleza |  | 28.01 (24.34-31.48) | 25.89 (20.50-31.28) | 29.95 (24.99-34.92) |  | 8.50 (6.05-10.95) | 7.79 (3.97-11.61) | 9.03 (5.85-12.22) |
| Goiânia |  | 27.67 (23.73-31.58) | 31.13 (24.46-37.81) | 24.75 (20.20-29.29) |  | 7.28 (4.86-9.70) | 6.51 (3.00-10.02) | 7.90 (4.57-11.23) |
| João Pessoa |  | 29.14 (25.00-33.27) | 26.82 (20.35-33.28) | 31.02 (25.78-36.27) |  | 6.97 (4.55-9.40) | 5.61 (2.06-9.16) | 8.07 (4.83-11.32) |
| Macapá |  | 29.98 (24.87-35.08) | 27.74 (20.24-35.24) | 32.21 (25.36-39.07) |  | 10.78 (6.93-12.62) | 12.91 (4.83-20.99) | 9.31 (5.59-13.03) |
| Maceió |  | 28.68 (24.60-32.67) | 23.56 (17.59-29.53) | 32.92 (27.55-38.29) |  | 9.05 (6.21-11.90) | 6.29 (2.55-10.03) | 11.09 (7.05-15.13) |
| Manaus |  | 35.77 (31.00-40.55) | 38.27 (30.44-46.11) | 33.34 (27.67-39.01) |  | 11.54 (8.04-15.04) | 14.62 (7.34-21.89) | 9.40 (6.10-12.70) |
| Natal |  | 30.95 (26.90-35.01) | 30.25 (23.64-36.86) | 31.56 (26.65-36.48) |  | 9.11 (6.10-12.11) | 10.23 (4.07-16.39) | 8.35 (5.43-11.28) |
| Palmas |  | 23.80 (19.79-27.80) | 24.42 (18.21-30.63) | 23.10 (18.03-28.16) |  | 7.42 (4.69-10.15) | 9.05 (4.16-13.93) | 6.03 (3.01-9.05) |
| Porto Alegre |  | 31.41 (27.29-35.52) | 36.05 (28.88-43.22) | 27.74 (23.06-32.41) |  | 10.92 (7.74-14.10) | 12.15 (6.10-18.20) | 10.19 (6.64-13.74) |
| Porto Velho |  | 26.65 (25.02-34.27) | 34.80 (26.97-42.62) | 24.59 (19.64-29.54) |  | 9.31 (5.87-12.74) | 10.30 (3.78-16.83) | 8.65 (5.19-12.11) |
| Recife |  | 32.19 (28.23-36.16) | 28.88 (22.76-30.00) | 34.65 (29.54-39.77) |  | 8.57 (6.04-11.10) | 7.81 (3.37-12.25) | 9.10 (6.08-12.12) |
| Rio Branco |  | 32.82 (29.54-39.77) | 33.46 (25.50-41.41) | 32.18 (26.20-38.17) |  | 10.31 (6.90-13.71) | 11.81 (5.03-18.58) | 9.50 (5.84-13.17) |
| Rio de Janeiro |  | 32.15 (28.33-35.96) | 29.48 (23.54-35.43) | 34.09 (29.18-38.99) |  | 10.58 (7.82-13.34) | 6.43 (3.20-9.67) | 14.06 (9.90-18.22) |
| Salvador |  | 27.64 (23.98-31.30) | 28.50 (21.93-35.08) | 27.14 (22.83-31.44) |  | 8.83 (6.27-11.40) | 8.73 (3.29-14.17) | 9.26 (6.39-12.13) |
| São Luís |  | 26.53 (22.00-31.06) | 27.44 (19.54-35.35) | 25.62 (20.41-30.82) |  | 6.59 (3.38-9.81) | 7.24 (-0.08-15.96) | 5.94 (3.16-8.72) |
| São Paulo |  | 29.25 (25.84-32.87) | 25.39 (20.33-30.46) | 32.48 (27.96-37.00) |  | 10.02 (7.43-12.61) | 8.36 (4.43-12.29) | 11.23 (7.81-14.64) |
| Teresina |  | 26.49 (22.56-30.42) | 27.63 (21.43-33.84) | 25.39 (20.38-30.39) |  | 7.88 (5.23-10.53) | 7.79 (3.53-12.06) | 7.98 (4.59-11.37) |
| Vitória |  | 25.67 (22.16-29.17) | 20.95 (15.93-25.97) | 26.64 (24.92-34.92) |  | 9.62 (6.74-12.50) | 6.61 (2.72-10.49) | 12.02 (8.03-16.01) |
| Distrito Federal |  | 29.53 (24.47-34.58) | 30.68 (22.40-38.46) | 28.33 (22.16-34.51) |  | 7.29 (4.44-10.13) | 8.27 (2.90-13.64) | 6.80 (3.52-10.07) |
| **Brazil** |  | **29.6 (28.4-30.7)** | **28.5 (26.7-30.4)** | **30.3 (28.8-31.8)** |  | **9.3 (8.5-10.1)** | **8.5 (7.1-9.8)** | **9.9 (8.9-11.0)** |

**Table S2:** Predictive accuracy of the model used to project BMI categories in Brazilian adults by 2030

| **Category** | **year** | **Lower CI** | **Upper CI** | **Predicted prevalence** | **Observed prevalence** | **MSE** | **MAE (mean absolute error)** | **Coverage Probability** | **10% Relative Error** | **5% Relative Error** |
| --- | --- | --- | --- | --- | --- | --- | --- | --- | --- | --- |
| Underweight and normal weight | 2014 | **48.12** | **48.65** | **48.38** | 45.32 | 9.39 | 3.06417 | 0 | 1 | 0 |
| Underweight and normal weight | 2015 | **47.04** | **47.63** | **47.33** | 44.29 | 9.25 | 3.0414 | 0 | 1 | 0 |
| Underweight and normal weight | 2016 | **45.95** | **46.61** | **46.28** | 43.81 | 6.09 | 2.46726 | 0 | 1 | 0 |
| Underweight and normal weight | 2017 | **44.85** | **45.60** | **45.22** | 43.95 | 1.62 | 1.27268 | 0 | 1 | 1 |
| Underweight and normal weight | 2018 | **43.75** | **44.59** | **44.17** | 43.09 | 1.16 | 1.0786 | 0 | 1 | 1 |
| Underweight and normal weight | 2019 | **42.65** | **43.58** | **43.12** | 42.66 | 0.21 | 0.45596 | 1 | 1 | 1 |
| Pré-obesity | 2014 | **33.82** | **34.32** | **34.07** | 36.03 | 3.84 | 1.95839 | 0 | 1 | 0 |
| Pré-obesity | 2015 | **34.19** | **34.75** | **34.47** | 36.62 | 4.62 | 2.14962 | 0 | 1 | 0 |
| Pré-obesity | 2016 | **34.54** | **35.17** | **34.86** | 36.89 | 4.14 | 2.03447 | 0 | 1 | 0 |
| Pré-obesity | 2017 | **34.87** | **35.58** | **35.23** | 36.74 | 2.29 | 1.51363 | 0 | 1 | 1 |
| Pré-obesity | 2018 | **35.18** | **35.99** | **35.58** | 36.9 | 1.74 | 1.31782 | 0 | 1 | 1 |
| Pré-obesity | 2019 | **35.47** | **36.38** | **35.92** | 36.67 | 0.56 | 0.74768 | 0 | 1 | 1 |
| Obesity class I | 2014 | **12.54** | **12.88** | **12.71** | 13.58 | 0.76 | 0.8736 | 0 | 1 | 0 |
| Obesity class I | 2015 | **12.95** | **13.33** | **13.14** | 14.01 | 0.76 | 0.87135 | 0 | 1 | 0 |
| Obesity class I | 2016 | **13.36** | **13.80** | **13.58** | 14.25 | 0.45 | 0.67151 | 0 | 1 | 1 |
| Obesity class I | 2017 | **13.77** | **14.28** | **14.03** | 14.18 | 0.02 | 0.15437 | 1 | 1 | 1 |
| Obesity class I | 2018 | **14.18** | **14.78** | **14.48** | 14.58 | 0.01 | 0.10022 | 1 | 1 | 1 |
| Obesity class I | 2019 | **14.60** | **15.28** | **14.94** | 14.99 | 0.00 | 0.04938 | 1 | 1 | 1 |
| Obesity class II and III | 2014 | **4.73** | **4.95** | **4.84** | 5.07 | 0.05 | 0.23218 | 0 | 1 | 1 |
| Obesity class II and III | 2015 | **4.94** | **5.18** | **5.06** | 5.09 | 0.00 | 0.03043 | 1 | 1 | 1 |
| Obesity class II and III | 2016 | **5.15** | **5.43** | **5.29** | 5.05 | 0.06 | 0.23872 | 0 | 1 | 1 |
| Obesity class II and III | 2017 | **5.36** | **5.69** | **5.53** | 5.14 | 0.15 | 0.38532 | 0 | 1 | 0 |
| Obesity class II and III | 2018 | **5.57** | **5.97** | **5.77** | 5.42 | 0.12 | 0.34943 | 0 | 1 | 0 |
| Obesity class II and III | 2019 | **5.79** | **6.25** | **6.02** | 5.68 | 0.12 | 0.3411 | 0 | 1 | 0 |
| **Overall predictive accuracy** |  |  |  |  |  | **1.98** | **1.06** | **21%** | **100%** | **54%** |

CI: confidence interval; MSE: mean square error.; MAE: mean absolute error

**Figure S1**: Projected prevalence of obesity and obesity class II and III in Brazil by 2030

Source: Figures were generated using Data wrapper (<https://www.datawrapper.de/>)
